# A continuum of executive function deficits in early subcortical vascular cognitive impairment: A systematic review and meta-analysis

**DOI:** 10.1590/1980-57642016dn11-040006

**Published:** 2017

**Authors:** Felipe Kenji Sudo, Patricia Amado, Gilberto Sousa Alves, Jerson Laks, Eliasz Engelhardt

**Affiliations:** 1 Departamento de Psicologia, Pontifícia Universidade Católica do Rio de Janeiro, RJ, Brazil.; 2 Instituto D'Or de Ensino e Pesquisa, Rio de Janeiro, RJ, Brazil.; 3 Instituto de Psiquiatria, Universidade Federal do Rio de Janeiro, RJ, Brazil.; 4 Departamento de Medicina Interna, Universidade Federal do Ceará, CE, Brazil.; 5 Goethe Universitat Frankfurt Am Main, Germany.; 6 Programa de Pós-Graduação em Biomedicina Translacional (BIOTRANS), Unigranrio, Duque de Caxias, RJ, Brazil.; 7 Setor de Neurologia Cognitiva e do Comportamento, INDC/CDA/ IPUB, Universidade Federal do Rio de Janeiro, RJ, Brazil.

**Keywords:** mild cognitive impairment, cerebrovascular disorders, neuropsychology, vascular dementia, metabolic syndrome, comprometimento cognitivo leve, transtornos cerebrovasculares, neuropsicologia, demência vascular, síndrome metabólica

## Abstract

**Background.:**

Subcortical Vascular Cognitive Impairment (SVCI) is a clinical continuum of vascular-related cognitive impairment, including Vascular Mild Cognitive Impairment (VaMCI) and Vascular Dementia. Deficits in Executive Function (EF) are hallmarks of the disorder, but the best methods to assess this function have yet to be determined. The insidious and almost predictable course of SVCI and the multidimensional concept of EF suggest that a temporal dissociation of impairments in EF domains exists early in the disorder.

**Objective:**

This study aims to review and analyze data from the literature about performance of VaMCI patients on the most used EF tests through a meta-analytic approach.

**Methods:**

Medline, Web of Knowledge and PsycINFO were searched, using the terms: “vascular mild cognitive impairment” OR “vascular cognitive impairment no dementia” OR “vascular mild neurocognitive disorder” AND “dysexecutive” OR “executive function”. Meta-analyses were conducted for each of the selected tests, using random-effect models.

**Results:**

Systematic review showed major discrepancies among the results of the studies included. Meta-analyses evidenced poorer performance on the Trail-Making Test part B and the Stroop color test by VaMCI patients compared to controls.

**Conclusion:**

A continuum of EF impairments has been proposed in SVCI. Early deficits appear to occur in cognitive flexibility and inhibitory control.

## RESUMO.

**Introdução:**

O Comprometimento Cognitivo Vascular Subcortical (CCVS) é um contínuo clínico de comprometimento cognitivo de causa vascular, que inclui o Comprometimento Cognitivo Leve Vascular (CCLV) e a Demência Vascular. Déficits em Função Executiva (FE) são marcantes no quadro, mas a melhor metodologia para avaliar essa função ainda necessita ser determinada. A evolução insidiosa e quase previsível do CCVS e o conceito atual multidimensional de FE sugerem que uma dissociação temporal de comprometimentos dos domínios da FE exista nos estágios iniciais do transtorno.

**Objetivo:**

Visou-se a revisar e analisar dados da literatura sobre o desempenho de sujeitos com CCLV nos testes mais usados de FE através de uma abordagem metanalítica.

**Métodos:**

Foram realizadas buscas no Medline, *Web of Knowledge* e PsycINFO, usando os seguintes termos: “comprometimento cognitivo vascular” OU “comprometimento cognitivo não demência vascular” OU “transtorno neurocognitivo leve vascular” E “função executiva” OU “disexecutiva”. Metanálises foram conduzidas para cada um dos testes, usando-se modelos de efeitos aleatórios.

**Resultados:**

A revisão sistemática demonstrou grande discrepância entre os resultados dos estudos incluídos. A metanálise evidenciou desempenhos piores no Teste de Trilhas parte B e no teste de cores do Stroop em sujeitos com CCLV em comparação com controles.

**Conclusão:**

Um contínuo de comprometimentos em FE no CCVS foi proposto. Déficits iniciais parecem ocorrer em flexibilidade cognitive e controle inibitório.

## INTRODUCTION

Subcortical Vascular Cognitive Impairment (SVCI), a clinical continuum of cognitive impairments due to small-vessel disease, has been recognized as a common cause of cognitive dysfunction and, ultimately, of dementia in the elderly population.^1^^,^ 2 Metabolic risk factors, such as diabetes mellitus, smoking and dyslipidemia, as well as systemic arterial hypertension, are assumed to promote continuous insults to the small perforating arteries, which may suffer occlusion or subocclusion due to progressive arteriolosclerosis, lipohyalinosis and fibrinoid necrosis.^3^ These vascular changes result in subcortical lesions: lacunar infarcts and white matter lesions, which can be detectable as punctuate periventricular white-matter hyperintensities (WMH) to extensive areas of leukoaraiosis on neuroimaging.^4^ These white matter lesions may impair deep (punctuate or confluent WMH on T2 and FLAIR images) and periventricular regions (WMH bordering the lateral ventricles).^5^

Small-vessel disease consistently disrupts interconnections among prefrontal, sensory, motor and limbic cortices, causing disturbances to complex cognitive functions dependent on the tight integration of high order decisional neurons in the prefrontal cortex with primary and association cortical areas.^6^ Among the affected abilities, impairment in executive function (EF) has been widely described in SVCI and resulted in its inclusion among the early cognitive changes associated with vascular-related neurocognitive disorders in the 5^th^ Edition of the Diagnostic and Statistical Manual of Mental Disorders (DSM-5).^7^

Although the relationship between SVCI and executive dysfunction is clear, some shortcomings regarding the best methods to assess EF still exist. The lack of harmonization on the choice of cognitive tests adopted in research has resulted in difficulties integrating and interpreting findings from different studies.^8^ To address these issues, the National Institute of Neurological Disorders and Stroke-Canadian Stroke Network Vascular Cognitive Impairment Harmonization Standards (NINDS-CSN) work group proposed, in 2006, specific neuropsychological protocols to evaluate cerebrovascular cognitive disorders.^8^ However, the long time required to apply these batteries has made their use unfeasible in most clinical settings.^9^ The same problem may affect the VADAS-cog, another proposed neuropsychological instrument for vascular-related cognitive impairment.^10^ In addition, EF has been increasingly recognized as a multidimensional rather than a unitary construct; the diverse range of abilities grouped under the umbrella-term of EF, such as cognitive flexibility, inhibitory control, working memory and “complex” unspecific EF, precludes direct comparison of results from different EF tests.^11^^,^ 12 Moreover, since small-vessel disease commonly exhibits an insidious course, which follows a largely predictable temporal pattern of subcortical lesions - from periventricular to juxtacortical regions - it follows that impairments in EF domains may also be subject to a temporal dissociation according to site of damage.^13^^,^ 14

Characterizing the EF changes in early SVCI could contribute toward a better understanding of the natural history of the disorder, thereby enabling early intervention to control vascular risk factors. This measure could help prevent the onset of Vascular Dementia.^15^ Furthermore, brief and widely accessible tests would be more suitable for clinical use than extensive sophisticated neuropsychological batteries. A recent systematic review has listed the most frequently used EF tests in studies, including the Trail-Making test part B (TMTB), the semantic and phonemic Verbal Fluency (sVF and pVF), the Clock Drawing Test (CDT), the Digit Span backwards (DSb), the Stroop test and the Wisconsin Card Sorting Test.^16^ The present study aims to review data on the performance of patients with early SVCI, namely Vascular Mild Cognitive Impairment (VaMCI), on these tests.

## METHODS

### Literature search.

Database searches were performed on Medline, Web of Knowledge and PsycINFO using the following combination of terms: “vascular mild cognitive impairment” OR “vascular cognitive impairment no dementia” OR “vascular mild neurocognitive disorder” AND “dysexecutive” OR “executive function”. No restriction on date of publication or field was placed. The reference lists of the selected articles were hand searched for potentially relevant papers.

The following inclusion criteria were applied for the selection of studies: (1) articles comparing performances on the most widely used EF tests (TMTB, sVF and pVF, STROOP test, DSb, CDT, and Wisconsin Card Sorting Test) between VaMCI patients and normal controls; (2) samples comprising older subjects ( ≥ 60 years old); (3) diagnosis of VaMCI based on the presence of cognitive impairments and relatively spared functional status and the presence of cerebrovascular disease; (4) cerebrovascular disease due to small-vessel disease and not associated with stroke or large-vessel disease; and (5) studies written in English, French, Spanish or Portuguese. Posters, case-reports, reviews, conferences and essays were excluded.

### Procedures.

Screening of the retrieved articles was carried out by two independent researchers (F.K.S. and E.E.). Data extraction was performed independently and discordant results were resolved through discussion with the whole team of authors.

### Quality assessment strategy.

The risk of biases in the selected studies was appraised through the following criteria derived from a published checklist.^17^

1. A representative sample of general elderly population was recruited with a minimum baseline participation rate of 70%;
2. Participation rate at follow-up was 70% of the baseline sample or greater (when applicable);
3. Follow-up duration was at least 1 year (when applicable);
4. Diagnosis of VaMCI was based on recognized international criteria by a consensus committee.^4^^,^ 7 ^,^ 18 ^,^ 19

Items 2 and 3 were not applied for cross-sectional studies. Samples were deemed representative of the overall elderly population if no limits for recruitment of participants regarding age (subjects were aged 60-65 years or older and no additional restrictions for age were adopted), gender, race, education and cognitive and clinical status (exclusion of subjects with dementia at baseline was accepted) were used. Studies which fulfilled these criteria were assigned as higher quality studies. Data derived from at least two higher quality studies was classified as “Grade 1 level of evidence”. “Grade 2 level of evidence” was attributed to single higher quality studies, whereas “inconsistent evidence” was that obtained from lower quality studies.

### Data synthesis.

Mean scores, standard deviations and sample sizes were extracted for each of the EF tests. These values were subjected to meta-analyses stratified by EF test. Data were assessed using R Statistics version 3.3.3. The Random effect approach was preferred over the fixed effect, so that weights of studies of different sizes could be balanced.^20^ Presence of heterogeneity across studies was identified through the determination of I.^2^

## RESULTS

**Figure 1:**
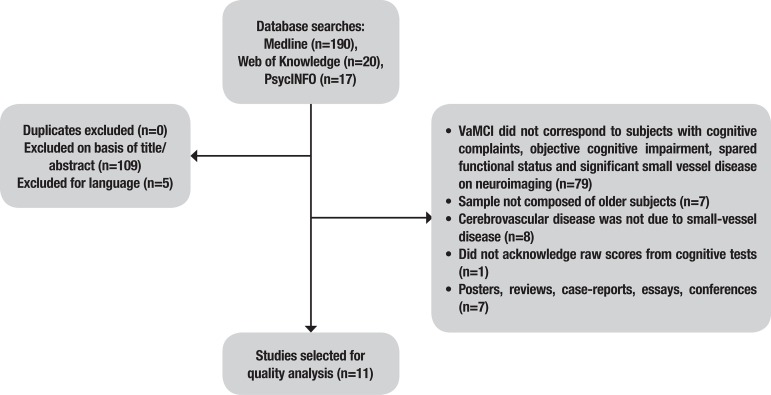
Stages for the selection of studies.

Eleven studies were selected from a total of 227 retrieved from the database searches. The stages for the selection of the studies are depicted in Figure 1.

### Samples.

A total of 849 individuals were included in the studies. Mean age of the samples was 72.5 ± 6.1 years and mean education was 10.2 ± 3.7 years. Female subjects represented at least 38% of the participants, although one of the studies did not state the number of individuals of each gender.^21^ Subjects were recruited at tertiary facilities in all but one study.^21^ Comparisons of sociodemographic variables indicated that normal controls were significantly younger than VaMCI subjects in two studies.^22^^,^ 23 Education was significantly higher in controls than in the VaMCI group in one article.^23^ Table 1 describes the sociodemographic characteristics of the selected studies.

**Table 1:** Sociodemographic characteristics of the samples.

Author, year	n*	Age (years)			Education (years)			Gender (n)	
		VaMCI	Controls		VaMCI	Controls		Male	Female
Garrett, 2004	43	78.4 ± 6.4	76.5 ± 8.9		13.6 ± 2.5	12.4 ± 3.6		21	22
Nordahl, 2005	28	77.6 ± 3.5	78.6 ± 6.3		13.5 ± 1.51	15.6 ± 2.8		8	20
Ishii, 2007	255	79.1 ± 6.9	72.4 ± 6.4		7.3 ± 2.2	8.3 ± 1.6		n.a.	n.a.
Gainotti, 2008	106	71.6 ± 5.9	70.9 ± 3.9		8.7 ± 4.4	9.3 ± 4.1		63	45
Seo, 2010	130	70.6 ± 6.4	67.7 ± 6.3		10.1 ± 4.8	10.8 ± 4.8		61	69
Bella, 2011	20	70.8 ± 6.3	67.7 ± 3.9		8.5 ± 5.2	10.1 ± 5.1		11	9
Marra, 2011	96	73.2 ± 6.2	70.9 ± 3.9		9.7 ± 4.9	9.3 ± 4.1		46	50
Fernández, 2011	38	72.2 ± 7.6	70.3 ± 8.1		3.6 ± 3.5	5.6 ± 2.6		16	22
Villeneuve, 2011	48	73.4 ± 5.1	71.0 ± 6.2		12.4 ± 5.2	12.7 ± 3.7		18	30
Chin, 2012	59	71.9 ± 6.3	66.6 ± 4.7		10.4 ± 4.9	13.5 ± 3.1		19	40
Sudo, 2013	26	74.1 ± 8.0	69.3 ± 7.1		8.8 ± 4.0	11.2 ± 2.5		9	17

* Number of controls + VaMCI; n.a.: not acknowledged; VaMCI: Vascular Mild Cognitive Impairment.

### Diagnosis of VaMCI.

Some variations in the diagnostic criteria used to identify subjects with VaMCI were detected among studies. Modified versions of Petersen`s criteria for Mild Cognitive Impairment (MCI)^24^ were adopted in five of the studies.^22^^,^ 25 ^-^ 28 In five of the articles, the detection of memory impairment was mandatory for the diagnosis of MCI.^21^^,^ 25 ^,^ 26 ^,^ 29 ^,^ 30 The criteria proposed by Frisoni (2002) for VaMCI^31^ was adopted by one of the studies, which included the presence of dysexecutive syndrome, memory deficits and functional preservation as necessary features for the diagnosis.^32^

Specific cutoff values in tests were applied to determine objective cognitive impairment: cognitive scores lower than 1.5 standard-deviations (16^th^ percentile) from normative data^22^^,^ 23 ^,^ 27 ^,^ 28 or at least two scores below established cutoff points in episodic memory tasks were employed in studies.^26^ Absence of functional impairment was determined through scores on structured questionnaires, such as the Pfeffer Functional Activities Questionnaire,^28^ the Functional Autonomy Measurement System^27^ and the Seoul Instrumental Activities of Daily Living scale.^23^

Subjects were classified as MCI if they had Clinical Dementia Rating scores of 0.5 in two studies.^21^^,^ 29 For the present review, in Ishii et al. (2007), normal controls were defined as subjects with CDR = 0 and without cerebrovascular disease, whereas VaMCI patients were defined as those with CDR = 0.5 and non-strategic infarcts.^21^

The presence of cerebrovascular disease was determined by the identification of focal neurological symptoms or signs and of severe white-matter abnormalities on neuroimaging.^22^^,^ 23 ^,^ 25 ^,^ 29 ^,^ 32 Some studies defined the presence of small-vessel disease in VaMCI according to the extension of WMH: infarcts less than 2 cm in size,^26^ lesions of at least 4 mm^21^ or the presence of WMH affecting over 19.375% of the total white-matter volume.^25^ Neuroimaging criteria defined by Erkinjuntti et al. (2000), comprising the presence of severe WMH (periventricular WMH > 10 mm and deep WMH ≥ 25 mm in maximum diameter) or moderate WMH with at least 5 lacunes,^33^ was adopted by two studies.^22^^,^ 34 The presence of moderate WMH, as indicated by scores > 1 on the modified-Fazekas Scale, and the absence of hippocampal atrophy, defined as scores on De Leon's scale of ≤ 1, were considered indicative of pure cerebrovascular disease in one study.^28^ Presence of confluent WMH defined the presence of vascular burden in one study.^27^

### Performance on EF tests.

Table 2 summarizes the scores on EF assessments of normal controls and VaMCI. The choice of EF tests varied greatly among studies, ranging from single screening tasks^25^ to long batteries.^26^^,^ 30 Since the Clock-Drawing Test was only included in one of the selected studies,^28^ it has not been shown in the table. The Wisconsin Card Sorting Test was not used in any of the included articles.

**Table 2:** Comparison of performances on EF tasks between Vascular Mild Cognitive Impairment patients and controls in the selected studies.

Study	n	TMTB				pVF				sVF				DSb				Stroop color test (seconds)		
		VaMCI	NC	p–value		VaMCI	NC	p–value		VaMCI	NC	p–value		VaMCI	NC	p–value		VaMCI	NC	p–value
Garrett, 2004	VaMCI = 18; NC = 25	190.5 ± 76.3	90.8 ± 33.5	<.0001		31.8 ± 9.6	29.4 ± 8.4	n.s.		14.3 ± 4.1	17.8 ± 6.2	n.s.		–	–	–		–	–	–
Nordahl, 2005	VaMCI = 10; NC = 17	–	–	–		–	–	–		12.6 ± 4.2	16.6 ± 3.9	.03		–	–	–		–	–	–
Ishii, 2007	VaMCI = 21; NC = 234	346.9 ± 122.1	221.4 ± 103.6	n.a.		–	–	–		6.2 ± 1.5	7.7 ± 2.4	n.a.		–	–	–		–	–	–
Gainotti, 2008	VaMCI = 41; NC = 65	–	–	–		23.2 ± 8.43	24.5 ± 9.6	.63		14.6 ± 3.8	14.9 ± 3.7	.84		3.3 ± 0.81	3.9 ± 1.0	.01		70.3 ± 26.5	53.7 ± 16.5	.001
Seo, 2010	VaMCI = 34; NC = 96	–	–	–		14.0 ± 7.6	26.4 ± 11.2	<.05		11.2 ± 4.5	16.5 ± 4.2	<.05		3.4 ± 1.0	3.7 ± 1.1	n.s.		–	–	–
Marra, 2011	VaMCI = 36; NC = 60	–	–	–		25.2 ± 10.3	24.4 ± 9.6	.91		14.9 ± 4.3	14.9 ± 3.8	.86		3.6 ± 1.2	3.9 ± 1.35	.096		66.8 ± 29.5	53.7 ± 16.5	.017
Bella, 2011	VaMCI = 10; NC = 10	–	–	–		–	–	–		–	–	–		–	–	–		41.1 ± 15.9	26.3 ± 11.8	<.05
Fernández, 2011	VaMCI = 19; NC = 19	–	–	–		–	–	–		12.1 ± 2.8	16.1 ± 2.2	<.05		3.1 ± 0.7	3.1 ± 0.5	n.s.		–	–	–
Villeneuve, 2011	VaMCI = 21; NC = 27	–	–	–		–	–	–		–	–	–		–	–	–		38.3 ± 11.4	27.5 ± 7.9	<.05
Chin, 2012	VaMCI = 31; NC = 28	–	–	–		17.0 ± 8.7	33.8 ± 8.4	<.05		23.2 ± 7.4	39.6 ± 6.9	<.05		3.5 ± 0.9	4.3 ± 1.0	<.05		–	–	–
Sudo, 2013	VaMCI = 15; NC = 11	265.8 ± 136.4	127.4 ± 46.7	.004		–	–	–		15.7 ± 4.5	16.6 ± 3.4	.80		–	–	–		–	–	–

VaMCI: Vascular Mild Cognitive Impairment; NC: Normal Controls; n.s. : not significant; n.a. : not acknowledged; TMTB: Trail-Making Test Part B; pVF: Phonemic Verbal Fluency; sVF: Semantic Verbal Fluency; DSb: Digit Span Backwards.

### Trail-Making Test B.

VaMCI subjects performed significantly worse than controls in two studies.^28^^,^ 29 A significant number of participants could not complete the TMTB in Fernandez et al. (2011) due to low education and results on the test were not analyzed by the authors.^32^ The presence of significant differences in time required to complete the TMTB between controls and VaMCI was not acknowledged in Ishii et al. (2007).^21^

### Verbal Fluency.

Controls performed significantly better than VaMCI patients on the sVF in some studies,^22^^,^ 23 ^,^ 25 ^,^ 32 whereas these differences were not found in other articles.^26^^,^ 28 ^-^ 30Most of the studies used the sum of words beginning with F, A and S as the method for calculating performance on the pVF task. VaMCI patients performed poorer than controls on the pVF,^22^^,^ 23 but these findings were not replicated in other studies.^26^^,^ 29 ^,^ 30 Different application methods for the VF were adopted in some studies, such as sVF using categories of objects found in a supermarket^22^ and pVF tests involving words beginning with the letter P (Fernández et al., 2011). The presence of significant differences in sVF scores between controls and VaMCI patients were not acknowledged in Ishii et al. (2007).^21^

### Stroop test.

The number of correct items during the reading (Stroop word test) and the inhibiting (Stroop color test) tasks were measured in two of the studies,^22^^,^ 23 while time for completion of these tasks was computed in other studies.^26^^,^ 27 ^,^ 30 ^,^ 34 Some authors included the number of errors during the color task as an additional measurement of inhibitory control.^26^^,^ 30 ^,^ 34 No significant difference was identified between controls and VaMCI subjects in the reading test, but time to complete the color reading was significantly higher in VaMCI subjects than controls in three of the studies.^26^^,^ 27 ^,^ 34 The number of errors was significantly higher in VaMCI patients compared to controls.^30^^,^ 34

### Digit Span backwards.

Controls performed significantly better than VaMCI participants in one of the studies.^23^ On the other hand, no significant differences were identified between VaMCI subjects and controls in the other articles which used this test.^22^^,^ 26 ^,^ 30 ^,^ 32

### Clock Drawing Test.

No difference was identified between controls and VaMCI patients on the clock drawing test, measured according to the CLOX method.^28^

### Risk of bias.

None of the selected studies had higher quality according to the criteria used in this review. Most of the studies recruited unrepresentative samples drawn from tertiary facilities. In all cases, diagnoses of VaMCI were based on different criteria from those determined by teams of specialists, or were highly dependent on performances on screening tests (e.g., MMSE) or global assessment scales (e.g., CDR). In some of the studies, only amnestic MCI subjects were included.^21^^,^ 25 ^,^ 29 ^,^ 30

### Meta-analysis.

Mean scores on EF tests were combined in a meta-analytic approach. Analyses were performed for each EF test.

**Figure 2:**
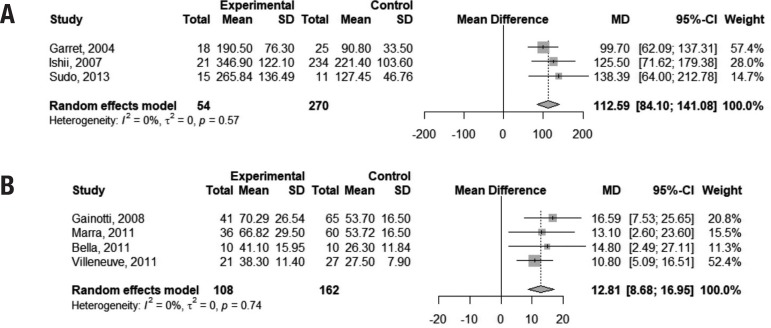
Forest plots assessing pooled scores on the TMTB (2a) and Stroop test (2b).

For the completion of the TMTB, pooled-analysis indicated that VaMCI subjects performed 112.59 seconds slower than normal controls (95% CI 84.10,141.08). Time to complete the Stroop color test was 12.81 seconds higher in VaMCI patients than controls (95% CI 8.68, 16.95). The I^2^ of 0% indicated absence of heterogeneity among studies for both tests (Figure 2).

**Figure 3:**
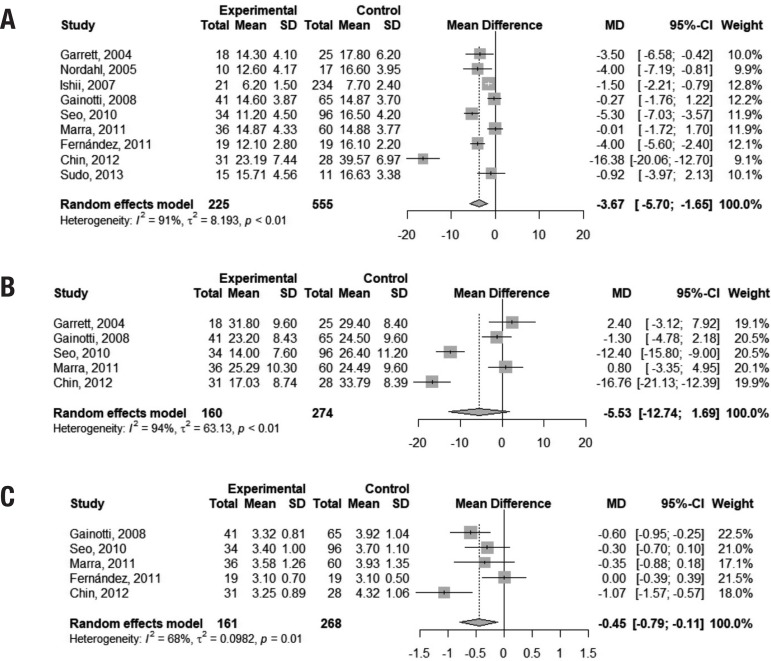
Figure 3. Forest plots assessing pooled scores on the Semantic Verbal Fluency task (3a), Phonemic Verbal Fluency task (3b) and Digit-Span Backwards test (3c).

Performance on the sVF (animals) was slightly worse in VaMCI subjects than in controls, with a difference of 3.67 points favoring the latter group (95% CI –5.70, –1.65). However, the presence of heterogeneity was marked among studies (I^2^ = 91%). Pooled analysis of scores on the pVF (letters FAS) showed inconclusive results (95% CI -12.74, 1.69). A very small mean difference was found in performances on the DSb between controls and VaMCI subjects (mean difference = –0.45 points; 95% CI –0.79, –0.11), but heterogeneity of studies was significant (I^2^ = 68%). Figure 6 depicts this result. Figure 3 illustrates these findings.

## DISCUSSION

Based on the present meta-analysis, in which we assessed performance of VaMCI subjects and normal controls on the most used EF tests in research, we suggest that a temporal dissociation of cognitive impairments may exist in SVCI. Results for at least two of the cognitive instruments have shown differences between early SVCI and healthy older subjects, with no significant heterogeneity among the studies. Most remarkably, data suggested a marked increase in the time required to complete the TMTB among VaMCI patients compared to normal healthy subjects. A slightly shorter time to perform the Stroop color test was also identified in controls relative to VaMCI individuals. On the other hand, results on the DSb, pVF and sVF tasks were either not significant or the studies were too heterogeneous to allow conclusions to be drawn.

Executive dysfunction has been regarded as the distinctive cognitive marker of SVCI by many authors.^35^^-^ 37 The EF tests analyzed in this study were those considered the most commonly used in research.^16^ Factor analyses and regression analyses studies indicated that they measure different aspects of EF. According to one of these studies, TMTB was identified as an index of cognitive flexibility.^38^ Difficulties on the Stroop color test have been associated with inhibitory control impairment.^39^ Moreover, multiple regression analyses indicated that sVF assesses semantic memory and working memory, whereas pVF is dependent on episodic verbal memory and cognitive speed.^40^

When individual EF domains were analyzed in subjects with Vascular Dementia, data in the literature suggested that all of them exhibited impairment when compared to controls.^35^^,^ 36 The present study suggests that specific EF domains, namely cognitive flexibility and inhibitory control, might be impaired in early SVCI (in the VaMCI stage), while other domains, such as working memory, might be initially preserved. Evidence from functional neuroimaging and pathology studies might support this idea. For instance, periventricular white matter receives blood supply from terminal vessels of long perforating branches of a watershed circulation (areas in which branches of the different cerebral large vessels meet). Most long perforating arteries are very tortuous and these anatomical characteristics make those locations especially vulnerable to hypoperfusion due to arteriosclerosis.^41^^,^ 42 Therefore, it could be predicted that periventricular WMH occur early in SVCI, because of chronic insults to the small vasculature associated with metabolic risk factors. Juxtacortical white matter, on the other hand, could be considered less susceptible to vascular damage and expected to be impaired later in the disorder.^13^^,^ 41 Because of this temporal-anatomical dissociation in white-matter lesions, neuronal pathways with periventricular trajectories, which include long interlobar fibers, could be disrupted in initial SVCI, whereas short corticocortical juxtacortical U-fibers might still be preserved in this stage.^13^^,^ 14 ^,^ 41 This mechanism might account for the continuum of EF impairments in SVCI, with earlier difficulties observed in abilities dependent on long fibers with periventricular trajectories, while other functions associated with short juxtacortical U-fibers might be subject to deficits later in the disorder.^14^

One study which evaluated neural correlates of TMTB scores in a post-stroke sample suggested that poor performance could be predicted by lesions situated in the lateral cholinergic pathways and in the left superior longitudinal fasciculus.^43^ In accordance, one fMRI study demonstrated that during the TMTB, left frontal and parietal areas, associated with motor, attentional, decisional, linguistic and sensory functions, are activated.^44^ Interlobar integration is also required during the Stroop color test, which may demand increased activity in frontal, parietal and occipital areas.^45^ On the other hand, a meta-analysis indicated that tasks which measure verbal working memory are mostly dependent on activation of the left dorsolateral prefrontal cortex.^46^ Also, another study showed that deep, but not periventricular WMH, correlated with working memory impairment.^47^ These data might explain the findings of early impairments in cognitive flexibility and inhibitory control in VaMCI, which we theorize might occur due to increased vulnerability to interlobar disconnection due to periventricular WMH in these cases. In addition, according to this hypothesis, working memory, which relies on the integrity of specific prefrontal areas, might be initially spared in SVCI.

The strength of the present study was allowing the identification of the EF domains affected in early SVCI, namely, cognitive flexibility and inhibitory control, through statistical combination of results from studies. This was not the case for the systematic review, given that major disparities exist among the selected articles, as revealed in this study. Moreover, assessment of the heterogeneity of the studies, performed by a meta-analytic method, raises questions over whether data derived from different sources are comparable or not. For this reason, conclusions drawn in a previous review on the cognitive correlates of white matter lesions in non-demented subjects should be considered with caution due to the selection of studies with potentially heterogeneous samples and methods.^48^ In addition, the current concept that EF comprises multiple distinct abilities and not a unitary entity may preclude direct comparisons between different EF tasks, which might have been a problem in a previous meta-analysis on the theme.^49^

Limitations of this study should be acknowledged. The quality assessment of the selected articles showed that risk of selection or diagnostic biases were significant in all. Since diagnostic criteria for VaMCI have been evolving over the years, differences in the characterization of this disorder varied greatly. For instance, some studies included only subjects presenting memory deficits, while others identified the cases based on the Clinical Dementia Rating of 0.5. Moreover, detection of cerebrovascular disease adopted different criteria, which might have led to variations in the severity of brain lesions among samples from different studies. Also, results on performances of VaMCI subjects in sVF, pVF and DSb were inconclusive due to high levels of heterogeneity among studies, despite the random effect models applied. Only one of the selected studies used the CDT and none employed the Wisconsin Card Sorting Test, hence, data on these tests remain unavailable. Finally, a low number of studies was selected and included small sample sizes, indicating that further research in this field is still needed.

Early identification of SVCI is crucial to allow intervention to control vascular risk factors before the onset of dementia. The hypothesis of a temporal continuum of dysexecutive syndrome, based on a multidimensional concept of EF and on pathophysiological aspects of lesion progression in SVCI, might be of great value for this purpose. However, further studies are needed to validate these theories.
